# The Enigma of Vasculitis

**DOI:** 10.1155/2010/312159

**Published:** 2010-06-10

**Authors:** Daniela Ghetie, Alla Rudinskaya, Raymond Raut

**Affiliations:** ^1^Department of Medicine (Rheumatology and Nephrology & Hypertension), Danbury Hospital, Danbury, CT 06810, Valhalla, NY 10595, USA; ^2^New York Medical College, Valhalla, NY 10595, USA

## Abstract

We describe the case of an 86-year-old man presenting with clinical symptoms suggestive of Temporal Arteritis. The evolution of the case prompted extensive work-up, including temporal artery and kidney biopsy. Based on the clinical and pathological findings, a diagnosis of ANCA-negative granulomatous necrotizing vasculitis involving the small-, medium-, and large-size vessels was made. The patient was treated with prednisone and cyclophosphamide, which was later switched to rituximab. The patient remained asymptomatic under this regimen and stabilization of his kidney function was achieved.

## 1. Introduction

Physicians have been continuously trying to find a way to better classify vasculitides in order to facilitate a fast and accurate diagnosis. Proper diagnosis is extremely important, as some diseases only require supportive treatment while others need prompt and aggressive therapy if significant morbidity and mortality is to be avoided. The clinical picture usually correlates with the size and extent of vessel involvement. The difficulty comes when physicians encounter complex cases that do not follow any current classification. 

Vasculitis of the temporal artery was observed in the biopsy specimen of a patient with clinical symptoms suggestive of Temporal Arteritis. Progressive renal dysfunction ensued despite rapid initiation of steroid therapy, prompting renal biopsy.

## 2. Case Report

86-year-old white man was referred to our rheumatology clinic on July 23rd, 2008. He was complaining of persistent fatigue, low grade fever, proximal arthralgias, and myalgias, especially in his hips. He had poor oral intake and weight loss. A PPD test done as outpatient was negative. His inability to eat was attributed to jaw claudication. Patient denied any vision changes, headaches or scalp tenderness. There was no skin rash or sicca symptoms.

Laboratory evaluation revealed an ESR of 64 mm/H, CRP 167.2 mg/L, WBC 12600/cumm, Hb 10.6 g/dL, Ht 32.3%, ferritin 2217 ng/mL, albumin 2.6 g/dL, BUN 18 mg/dL, and Cr 0.87 mg/dL.

A diagnosis of polymyalgia rheumatica and giant cell arteritis was suspected and treatment with prednisone 60 mg daily was started. Temporal artery biopsy performed in July of 2008 revealed granulomatous vasculitis with fibrinoid necrosis. The patient had dramatic improvement of his symptoms and rapid decline of the inflammatory markers. On August 15th, 3 weeks after initiating steroid therapy, his ESR decreased to 39 mm/h and CRP to 21.7 mg/dL. Over the next three weeks the steroid dose was gradually reduced to 30 mg daily. However, he did not tolerate further taper due to recurrence of polymyalgia-like symptoms. 

Despite overall clinical improvement and continuous treatment with prednisone (20–30 mg daily), renal dysfunction ensued over the three months since the onset of the patient's illness. In September, 2008 patient's BUN rose to 77 mg/dL and Cr to 3.33 mg/dL. Urinalysis revealed 2+ proteins, few RBCs, no RBC casts, and no WBC. At that point a vasculitic process in the kidneys was considered, prompting further workup. The following serologies were negative or normal: ANCA, C3, C4, CH50, ANA, anti-dsDNA, anti-GBM, anticardiolipin antibodies, and cryoglobulins. Hepatitis panel was negative. 

**Figure 1:**
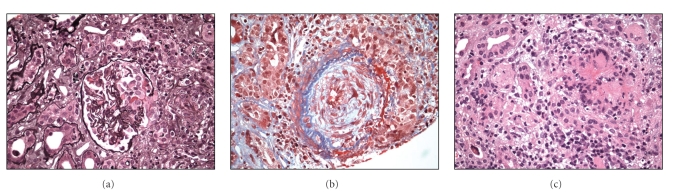
(a) Crescent formation. (b) Granuloma. (c) Vasculitis in medium-sized renal artery.

Magnetic Resonance Angiography was negative for renal artery stenosis. In September, 2008, the kidney biopsy was performed, showing acute and chronic granulomatous necrotising vasculitis of medium-sized (renal) arteries and crescent formation. The immunofluorescence report was negative for IgG, IgM, IgA, C3, C1, fibrinogen, albumin, or light chains (Figures 1(a), 1(b), and 1(c)). 

Based on the clinical and pathological findings, we concluded that our patient had ANCA-negative granulomatous necrotizing vasculitis involving the small-, medium- and large-size vessels.

In October, 2008, cyclophosphamide was added to prednisone. The regimen of cyclophosphamide 50 mg daily alternating with 75 mg daily and prednisone 15 mg daily was continued for four months. Of note, higher dose of cyclophosphamide was not tolerated due to severe leucopenia. This regimen achieved stabilization of the renal function to a creatinine of 2.3–2.5 mg/dL and normalization of the inflammatory markers, while no other symptoms were present. In February, 2009, cyclophosphamide was discontinued due to the concern for potential side effects and toxicity of cyclophosphamide. Patient was administered a course of rituximab, 1000 mg 2 doses 2 weeks apart with intention to prevent the disease flare upon stopping cyclophosphomide. Patient continued to taper the dose of prednisone gradually and discontinued it completely in June, 2009. As of March of 2010, he feels very well without any polymyalgia or cranial symptoms, resumed his regular tennis playing and other activities. His renal function has been stable with BUN of 35 mg/dL and creatinine of 1.64 mg/dL. Inflammatory markers, including ESR and CRP are normal.

## 3. Discussion

Classifying a vasculitis can be a real challenge and most likely the final word in the classification has not yet been said. The current approach using the vessel size (small, medium, and large) is useful in suggesting clinical features associated with a particular disease [1]. It is important to realize that there are unclassified vasculitic processes when clinical judgement and case particularities are guiding the best therapeutic approach. Vessels are very complex and not enough studied organs as determinants of a vasculitic pattern. More research is being oriented toward establishing the basis for this selective vulnerability beyond vessel size. It has been hypothesized that several factors, including embryogenesis, age-related changes, immunologic susceptibility, and injury-repair process might be involved in the etiology and pathogenesis of vasculitis [2]. 

A patient rarely presents with all of the classic findings [3]. Our patient presented with a clinical picture suggestive of Temporal-Arteritis. Temporal artery biopsy is a simple tool for diagnosis of vasculitis, but vasculitis of the temporal artery is not limited to Giant Cell Arteritis. Other systemic vasculitides (e.g., Polyarteritis nodosa, Wegener's granulomatosis, microscopic polyangiitis, Churg-Strauss Syndrome, Systemic Lupus Erythematosus) may show inflammatory changes on the temporal artery biopsy [4–8]. There have been case reports of systemic vasculitis (e.g.,Wegener granulomatosis), found to have biopsy-proven vasculitis of the temporal artery [9]. In 2003, Hamidou et al. reported seven cases of systemic necrotizing vasculitis with histologically proven temporal artery involvement [10].

In 1998, Lenz et al. described a patient with cranial symptoms suggestive of Temporal arteritis who was also diagnosed with ANCA-negative pauci-immune glomerulonephritis. No temporal artery biopsy was done in that patient. In our patient the biopsy of the temporal arteries showed granulomas and fibrinoid necrosis. Fibrinoid necrosis is atypical for giant cell arteritis and its presence should raise doubts about such a diagnosis [11]. 

Our patient's kidney biopsy was consistent with pauci-immune ANCA-negative granulomatous necrotizing glomerulonephritis. The rare finding of granuloma in the kidney biopsy, mandated the exclusion of other potential culprits beside the vasculitic etiology (most commonly Wegener's granulomatosis). The differential diagnosis of granulomatous renal disease is broad, including tuberculosis as a classic cause of noncaseating granuloma. Our patient had no risk factors for tuberculosis, negative PPD test, and no pulmonary symptoms whatsoever. We excluded also other potential infectious causes (e.g., fungal infections like histoplasmosis and cryptococcosis, chronic pyelonephritis, cat-scratch fever). There was no clinical or imagistic evidence of sarcoidosis, either. 

Chaveau et al. showed that renal involvement occurs in 75% of the patients with systemic necrotizing small-vessel vasculitis [12]. The absence or paucity of vascular immune deposits distinguishes microscopic polyangiitis, Wegener's granulomatosis (WG) and Churg-Strauss syndrome from the variety of necrotizing vasculitides.

The classic triad of respiratory tract granulomatous inflammation, systemic small-vessel vasculitis and necrotizing glomerulonephritis suggests the diagnosis of WG, but a lot of patients do not present with the classic findings. Antineutrophilic cytoplasmic antibody testing (ANCA) is a very important diagnostic aid, with 70% of the WG's having antibodies towards proteinase 3(PR3-ANCA), 20% against myeloperoxidase, while 10% may be ANCA negative. Approximately 80% of the patients with WG will have glomerulonephritis. The glomerulonephritis is characterized by focal necrosis, crescent formation, and the absence or paucity of immunoglobulin deposits. A similar pauci-immune, crescentic, necrotizing glomerulonephritis is found in patients with microscopic polyangiitis and Churg-Strauss Syndrome. The latter is characterized by a triad of asthma, eosinophilia and necrotizing granulomatous vasculitis. P-ANCA(or anti-MPO) is positive in 35%–75% of patients and up to 10% can have a positive C-ANCA. Microscopic polyangiitis is differentiated by the absence of asthma and development of rapidly progressive glomerulonephritis (RPGN) without granulomata. Over 80% of patients have ANCA, mostly perinuclear type.

Our case though, does not fit into any of the classic granulomatous necrotizing vasculitis.

When facing a systemic vasculitis it is extremely important to make an accurate diagnosis. As of yet we have no diagnostic criteria to differentiate vasculitis. A clinician has to take into consideration both the clinical manifestations and the histological findings, in his endeavors to understand these rare and devastating conditions [13]. 

Tissue-sampling is an invaluable tool in establishing the correct diagnosis. Aggressive, early, and appropriate treatment minimizes disease-related mortality and irreversible damage. Whereas steroid treatment is usually sufficient for Giant Cell Arteritis, renal involvement in the form of glomerulonephritis, traditionally requires addition of immunopsupressive drugs. Before the institution of immunosuppressive therapy, the mortality rate of patients with systemic vasculitis (such as WG or polyarteritis nodosa) was 75% at 5 months. When cyclophosphamide was administered along with corticosteroids, the 5-year mortality rate lowered to 12% [3]. The risks and benefits of aggressive immunosuppression must be assessed in each patient and treatment established accordingly.

Our case does not fall into any classifiable vasculitic profile. To our best knowledge, this is the only reported case of ANCA-negative systemic granulomatous necrotizing vasculitis involving small-, medium-, and large-size vessels in a patient facing the diagnosis of a systemic, yet unclassified vasculitis.
